# Synthesis of flower-like MnO_2_ nanostructure with freshly prepared Cu particles and electrochemical performance in supercapacitors

**DOI:** 10.1371/journal.pone.0269086

**Published:** 2022-06-02

**Authors:** Lingling Shen, Linghui Peng, Runfang Fu, Zichuan Liu, Xuchuan Jiang, Dexi Wang, Ali Reza Kamali, Zhongning Shi

**Affiliations:** 1 School of Mines, China University of Mining and Technology, Xuzhou, China; 2 Beijing Advanced Innovation Center for Materials Genome Engineering, Beijing Information Science & Technology University, Beijing, China; 3 Department of Chemical Engineering, Monash University, Clayton, Australia; 4 School of Chemical Equipment, Shenyang University of Technology, Liaoyang, China; 5 State Key Laboratory of Rolling and Automation, Northeastern University, Shenyang, China; Texas A&M University at Qatar, QATAR

## Abstract

Four types of flowerlike manganese dioxide in nano scale was synthesized via a liquid phase method in KMnO_4_-H_2_SO_4_ solution and Cu particles, wherein the effect of Cu particles was investigated in detail. The obtained manganese dioxide powder was characterized by XRD, SEM and TEM, and the supercapacity properties of MnO_2_ electrode materials were measured. The results showed that doping carbon black can benefit to better dispersion of copper particles, resulting in generated smaller size of Cu particles, and the morphology of MnO_2_ nanoparticles was dominated by that of Cu particles. The study of MnO_2_ synthesis by different sources of Cu particles showed that the size of MnO_2_ particles decreased significantly with freshly prepared fine copper powder compared with using commercial Cu powder, and the size of MnO_2_ particles can be further reduced to 120 nm by prepared Cu particles with smaller size. Therefore, it was suggested that the copper particles served as not only the reductant and but also the nuclei centre for the growth of MnO_2_ particles in synthesis process MnO_2_, and that is the reason how copper particles worked on the growth of flower-like MnO_2_ and electrochemical property. In the part of investigation for electrochemical property, the calculated results of b values indicated that the electrode materials have pseudo capacitance property, and the highest specific capacitance of 197.2 F g^-1^ at 2 mV s^-1^ and 148 F/g at 1 A/g were obtained for MCE electrode materials (MnO_2_ was synthesized with freshly prepared copper particles, where carbon black was used and dispersed in ethanol before preparation of Cu particles). The values of charge transfer resistance in all types of MnO_2_ materials electrodes were smaller than 0.08 Ω. The cycling retention of MCE material electrode is still kept as 93.8% after 1000 cycles.

## 1. Introduction

Recent years, environmental issues and diminishing supply of fossil fuels push us to put more efforts on the research of sustainable and renewable energy resources. Therefore, energy storage devices including electrochemical capacitors (ECs) and batteries have received intensive attentions, especially for ECs, processing higher power density and longer cycle life compared with batteries [1, 2]. Carbon, conducting polymers and transition metal oxides are the most widely investigated ECs electrode materials [3–8]. Among these types of ECs electrode materials, manganese dioxide (MnO_2_) has attracted significant interests for ECs electrode materials owing to the characters of high specific capacitance, low cost, abundance and environment friendly [9]. However, the most challenging issue for ECs devices facing today is to increase the energy density. Thus, seeking out the solution to this problem can help ECs move closer to batteries and cut down on the manufacture costs at the same time [10, 11]. These problems enable the nanomaterials, offering greatly improved ionic transport and electrical conductivity, to be the most promising candidate in energy storage field [12].

Currently, the guiding principle to solve the problems mentioned above is to form various composite structures, which can take fully advantages of the feature of every single component. The composite structures follow the strategy of the alliance of different components of active materials. Some scientists enhanced the electrochemical property with the help of graphene [13–15]. For example, Zhai et al. fabricated MnO_2_ on porous graphene gel/Ni foam(denoted as MnO_2_/G-gel/NF) with a large areal capacitance of 234.2 F g^-1^ and good rate capability [14]. This material worked as the positive electrode and the G-gel/NF worked as the negative electrode, resulting in the supercapacitor achieving a remarkable energy density of 0.72 mW h cm^-1^. Moreover, the combinations of metal oxides/ carbide, conducting polymers and MnO_2_ can improve the electrical conductivity and charge-discharge transport on the electrode surface and in the bulk materials effectively [16–21]. Rahmanabadi et al. prepared nickel–manganese dioxide and cobalt–manganese dioxide, and the specific capacitance values of 377 and 307 F g^-1^ were obtained at a high CV scan rate of 200 mV s^-1^ [13]. Ansari et al. investigated the electrochemical behavior of the fibrous polyaniline–MnO_2_ nanocomposite, and the results showed that a high capacitance of 525 F g^-1^ at the current density of 2 A g^-1^ was achieved [18]. This improved electrochemical behavior was attributed to the enhanced electrical conductivity caused by a larger surface area of the electrode and the electrolyte. In addition, the combinations of polymer, graphene and metal oxides with MnO_2_ make MnO_2_ based electrode materials more promising for energy storage [17, 22, 23]. Muhammad et al. fabricated MnO_2_@polymer/graphene nanocomposite electrode material by hydrothermal method, and the nanocomposite exhibited a specific capacitance of 1369 F g^-1^ at 3 A g^-1^ [22].

Much effort has been put to explore the application of MnO_2_ in energy storage field due to the large theoretical capacity of 1370 F g^-1^. Building the composite structure is an effective approach to improve the electrochemical property of MnO_2_, and several methods were widely used for the synthesis of MnO_2_ nanomaterials, including hydrothermal synthesis, electrochemical preparation and sol-gel method [24–29]. Besides, it is found that electrochemical performance can be improved under condition of high temperature and carefully technological design, however it consumes too much energy and needs complex operation steps during the preparation process [30–32]. For example, trio-composite was prepared at 400°C for 2 h in continuous nitrogen gas flow [33] and carbon decorated MnO_2_ was synthesized at temperature increasing up to 800°C under argon [34]. Hydrothermal method is much easier to operate at lower temperature and with a wide range of reagent candidates, which can be achieved in aqueous solution and organic solution [35–37]. Chemically liquid phase method can be operated at milder conditions compared with hydrothermal method, including normal temperature and pressure, and it was a more convenient method for synthesis of nanomaterials [38]. Based on this, our study will be devoted to synthesis of MnO_2_ via simple procedure and low energy consumption, and it is the novelty and the biggest difference in our research.

In this work, flower-like MnO_2_ nanostructure is synthesized with a facile one-step route at room temperature, where copper (Cu) particles work as reducing agent and the nuclei site of fabricated flower-like MnO_2_ nanostructure. MnO_2_ nanostructure with size of 120nm can be obtained by using 100 nm of copper particles. The specific capacitance of the MnO_2_ synthesized by prepared copper particles is higher than that of obtained by commercial copper. Besides, the electrochemical performance of prepared MnO_2_ structure was compared with the recently leading-edge reports, and it is suggested that the performance in this work is comparable with that of reports using simple MnO_2_ microstructure, however, the work of synthesis procedure and structure design still need to be improved for a better capacitance. The profound finding is that doping carbon black can fine size of synthesized powder. In this respect, a novel approach of adjusting morphology was proposed.

## 2. Experimental

### 2.1. Materials and reagents

Cu powder(<425μm) (99.5%), Cu(CO_2_CH_3_)_2_·H_2_O(98%), hydrazine hydrate (N_2_H_4_·H_2_O) (78–82%), NH_3_· H_2_O (28%), carbon black(Super P* Conductive, 99%), H_2_SO_4_ (98%) and KMnO_4_ (99.5%) were all purchased from Sigma (Australia). All of the chemicals were used as received without further treatment.

### 2.2. Synthesis of Cu powder and with carbon doped Cu powder

Cu nano powder was prepared via one-step chemical reduction at room temperature. In this process, 0.5mmol Cu(CO_2_CH_3_)_2_ was added into deionized (DI) water followed by magnetic stirring for 15min. After an even solution was obtained, 3ml NH_3_·H_2_O and 1ml N_2_H_4_·H_2_O were added into the solution with stirring for 15min and 60min, subsequently, and the Cu particles obtained via this way can be abbreviated as C.

For the carbon doped Cu powder fabrication, 0.002 g carbon black was added before addition of N_2_H_4_·H_2_O via two routes. One way was adding the carbon black into solution directly abbreviated as CCD, while the other way was dispersing the carbon black in ethanol (10 ml) in advance abbreviated as CCE. The products were washed successively by centrifuging and re-dispersion in ultrasonic bath with DI water and dehydrated ethanol several times. The Cu powder was dried under vacuum at 60°C for 4 h.

### 2.3. Synthesis of the MnO_2_ powder

Four types of the MnO_2_ powders were synthesized via four types of Cu particles and abbreviated as M, MC, MCD and MCE. M sample was obtained by using commercial Cu powder, and the other samples were synthesized by freshly prepared Cu particles, including C, CCD and CCE in section of 2.2. For synthesis of MnO_2_ powder, 0.0553 g KMnO_4_ and 3 ml H_2_SO_4_ (1 mol/L) were added into 35 ml DI water followed by magnetic stirring for 15 min. Then 0.0448 g of commercial Cu powder, prepared Cu powder, CCD powder and CCE powder were added into the above solutions, respectively, with magnetic stirring for 2 h. Washing and drying processes were the same as the fabrication of the Cu powder.

### 2.4. Preparation of the MnO_2_ electrode materials

First, 0.035 g of active material (MnO_2_ nanoparticles) and 1.2 mL NMP (1-Methyl-2-pyrrolidinone) were added into a small vial (vial A). Then, 0.01 g of carbon black (Super P) and 0.8 mL NMP were added into another small vial (vial B). In the next step, both vial A and vial B were ultrasonically treated for 1 h with an ultrasonic cleaner. Once finished, 0.005g of PVDF powder was added to vial B, and 0.05g powder totally with mass radio of 70%MnO_2_-20%CB-10%PVDF was used for the preparation of electrode materials. Then, vial B was treated in an ultrasonic cleaner for another hour. Next, the slurries in vial A and vial B were mixed, and the mixed slurry was treated in an ultrasonic cleaner for one more hour. The mixed slurry containing active material was then ready for use.

Several pieces of nickel foam (2cm×1cm×0.1cm) were put in 2 mol·L^-1^ HCl for 12 hours in standing to remove the oxide layer. Once finished, these nickel foams were thoroughly washed with water and acetone sequentially and dried in an oven. These nickel foam pieces were then folded in half along their length. Dividing by the indentation, a half of each nickel foam piece was squeezed using a hydraulic machine. The non-squeezed half of each was dipped and soaked into the slurry prepared previously. Next, the nickel foams soaked with slurry were transferred to a vacuum drying oven and heated at 110°C for 8 h. Once finished, the nickel foam pieces with active material were pressed into thin foils and ready to be used. The mass for samples was around 2mg, and the prepared electrode material the thickness fluctuated between 1.0 mm and 1.1 mm.

### 2.5. Electrochemical measurements

The measurements of electrochemical property were performed in 1 M Na_2_SO_4_ aqueous solution with a conventional three-electrode system, where the prepared samples (MnO_2_), Pt mesh(10 mm×10 mm) and the saturated Ag/AgCl electrode were served as working electrode, counter electrode and reference electrode, respectively. Electrochemical measurements including cyclic voltammetry (CV) (at scan rates ranging from 2 to 100 m*V* s^-1^), galvanostatic charge-discharge (GCD) studies (at current densities increasing from 1 to 40 A g-1) and electrochemical impedance spectroscopy (EIS) (at frequencies between 100 KHz and 10 mHz) were performed using an electrochemical work-station (PARSTAT 4000). The specific capacitance was calculated from cyclic voltammogram using the following formula:

$$C=\frac{(\int I\cdot dV)/\nu \cdot m\cdot V}{2}$$ (1)

Where *I* (A) is the charge-discharge current, ν (V/s) is scan rate, *m* is total mass (g) of electrode material, and *V* (V) is the applied potential window.

Specific capacitance based on GCD measurement was calculated using the following Eq (2), and for a linear discharge curve, it can be calculated with Eq (3):

$$C=\frac{2i_{d}\cdot \int \nu \;dt}{{\left(\Delta V\right)}^{2}}$$ (2)

$$C=\frac{I\cdot \Delta t}{m\cdot \Delta V}$$ (3)

where ∫*v* dt is the integral area of the discharging curves, *i*_d_ is the discharging current density, Δt is the discharging time and Δ*V* is the operating potential window.

### 2.6. Characterization

The phase composition of the product was analyzed by X-ray diffraction (XRD), a Rigaku Miniflex 600 diffractometer (Japan) with Cu Kα radiation (λ¼ = 1.5418 Å) at 40 kV, 15 mA and a scanning rate of 5°/min. The morphology and element distribution of the products were characterized by SEM (FEI Magellan, America) with EDS detector attached at an acceleration voltage of 5 kV and 15 kV, respectively. The morphology and microstructure of the synthesized particles were analyzed using transmission electron microscope (TEM, FEI Tecnai T20, America).

## 3. Results and discussion

### 3.1. Characterization of the fabricated MnO_2_

For the preparation of manganese dioxide powder, four typical products named M, MC, MCD and MCE are derived from reactions with commercial Cu powder, prepared Cu, CCD powder and CCE powder as shown in section of 2.2 and 2.3, respectively.

**Fig 1:**
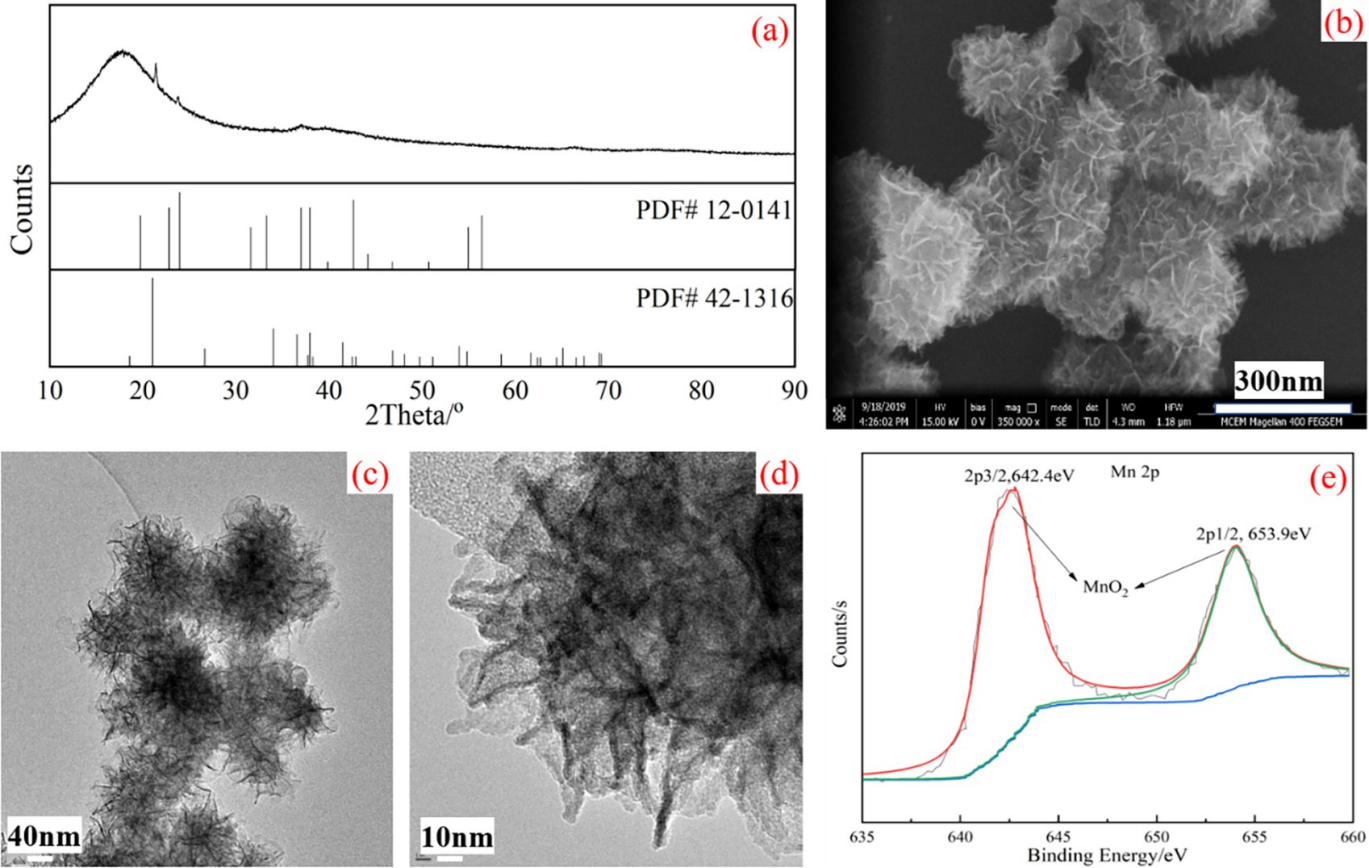
Characterization of the flower-like MCE particles: (a) XRD pattern, (b) SEM image, (c) TEM image at a low magnification, (d) TEM image at a high magnification, and (e) XPS spectrum.

The typical XRD pattern of MCE is shown in Fig 1(A), and it can be found that the prepared powder exhibits poor crystallinity. It is suggested that the powder may be composed of MnO_2_ (PDF#42–1316 and PDF#12–0141). A further phase identification with XPS analysis is shown in Fig 1(E), and it will be discussed later. However, the intensity of these peaks is low, which means that MCE is not well crystallized. It should be noted that although MnO_2_ powder was synthesized by using copper, the X-ray diffraction pattern shows no characteristic peaks of Cu particles. It means that all of the added Cu powder is consumed in the reactions. Two possible reaction routes are proposed as shown following reactions (1) and (2). Due to the amount of reactants, especially in strong acid condition, the reaction (1) tends to attribute to the formation of MnO_2_ phase in our synthesis process. Even though byproduct of Cu_2_O according to reaction (2), Cu_2_O could be converted to CuSO_4_ due to excess sulfuric acid.


$$3Cu+2KMnO_{4}+4H_{2}SO_{4}=3CuSO_{4}+2MnO_{2}+K_{2}SO_{4}+4H_{2}O$$ (1)

$$6Cu+2KMnO_{4}+H_{2}SO_{4}=3Cu_{2}O+2MnO_{2}+K_{2}SO_{4}+H_{2}O$$ (2)

The SEM image (Fig 1(B)) shows that the shape of MCE particles is flower-like, and the flower-like shape is composed of thin slices. The thickness of the slice is around 5nm, and the diameter of the flower is about 120nm. It can be observed from the TEM image (Fig 1(C)) that the distribution of the slices is very loose and a flower-like shape is formed. The TEM image at high magnification reveals that the slice is 4nm in thickness, which is in good agreement with SEM images. X-ray photoelectron spectroscopy (XPS) is used to further identify the composition of the product and is shown in Fig 1(E). The high resolution spectrum of Mn 2p is analyzed by XPS PEAK program. The fitted peaks at 653.9 eV and 642.9 eV are assigned to the Mn 2p orbital in the MnO_2_ phase, which can be a further evidence for that the product is manganese dioxide.

**Fig 2:**
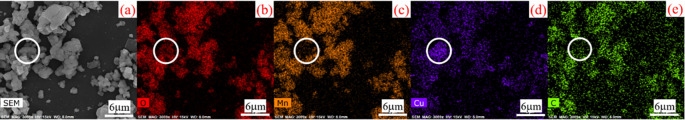
SEM image (a) and O (b), Mn (C), Cu (d) and C (e) elements mapping of the fabricated MCE powder.

In order to verify elements distribution of the fabricated MCE product, EDS mapping analysis was carried out and the results are shown in Fig 2, wherein color of orange, red and green represents for Mn, O and C element respectively. It can be found that Mn, O and C elements are well-distributed in the particles, indicating that carbon is doped and the product is composed of Mn and O elements. Besides, it should be noted that Cu element is also detected, but the intensity is weak. Thus, it is indicated that the content of copper compounds is lower compared with that of MnO_2_, suggesting that it is residual copper sulfate not Cu_2_O exist onto the surface of prepared powder. For Cu elemental mapping (blue color), the circular region shows strong color contrast, while the circular region of other elements shows weak color contrast. Especially for O elemental mapping, the existing O in circle area exhibits weaker contrast than other area, while in Mn elemental mapping, it shows no signal in the circle area. The results indicate that only few amount of copper materials exists in the prepared product, and it supposes to be Cu_2_O according to the reaction (2). The major procedure follows Eq (1) and pure MnO_2_ powder is obtained. But with the consumption of sulfuric acid and the insufficiency of ion diffusion, Cu_2_O is formed in certain sites based on reaction (2). Although the reaction (2) occurs, it just happens in some spot area, resulting in slight amount of Cu_2_O, and it can not be detected by XRD. Therefore, it is suggested that the formation of MnO_2_ follows the reaction 2 dominantly.

**Fig 3:**
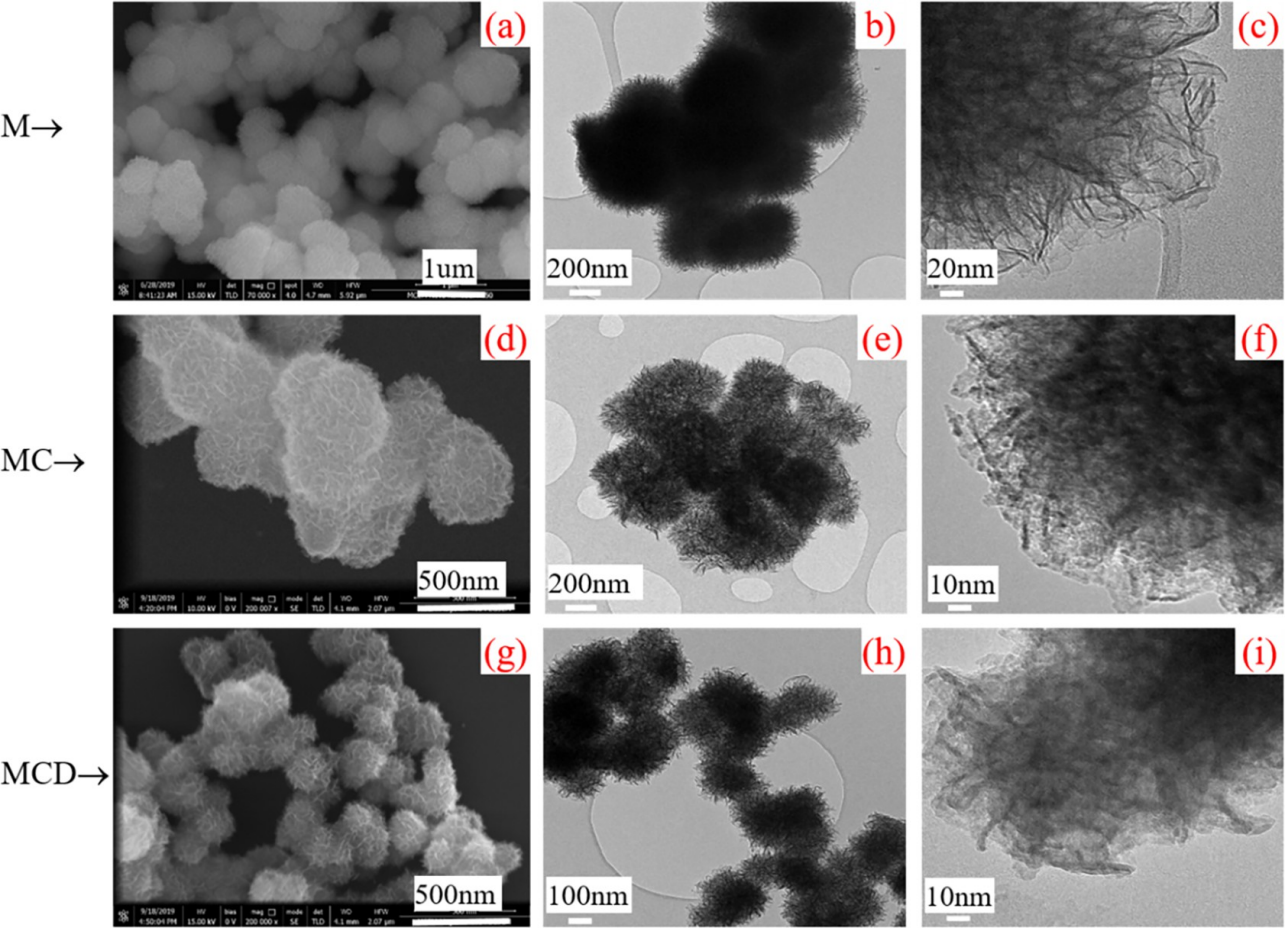
Characterization of produced manganese dioxide particles: (a) SEM image, TEM images at (b) a low magnification and (c) high magnification of product M; (d) SEM image, TEM images at (e) a low magnification and (f) high magnification of product MC; and (g) SEM image, TEM images (h) at a low magnification and (i) high magnification of product MCD.

For comparison, the other three types of MnO_2_ powder, including M, MC and MCD, are characterized by SEM and TEM, as shown in Fig 3.

It can be seen from the SEM image that product M are micro sized with flower shape, and particles are gathered together forming large clusters. The size of flower-like particles ranges from 0.6 μm to 1 μm. Compared with the product obtained by using commercial Cu particles, the size of particle MC decreases to 400 nm as shown in Fig 3(D). Fig 3(G) shows that the product MCD is more dispersed and the diameter of the flower shaped particles is only 200 nm.

From the TEM images shown in (Fig 3B, 3E and 3H), it can be observed that the product M has a poor dispersibility compared with the products MC and MCD, and a large size of 700 nm is obtained. However, the size of the fabricated MnO_2_ decreases significantly by using prepared copper particles, which can be found in (Fig 3E and 3H), and the details will be discussed later. The TEM images in Fig 3 show that the of four types of MnO_2_ nanostructure have the same slices width of 4 nm, which matches well with the SEM images in Fig 2(A).

By comparing the characterization results in Figs 2 and 3, it can be found that the morphologies of MCD and MCE are obviously different, although both are obtained by using doping carbon black in the synthesis of copper particles. The morphology of the product MCD is rambutan like and the edge is round, while the out edge of nano flower MCE is not as round as that of MCD. The gap between the slices in MCE is much larger than that of in MCD, which maybe owed to the distinctive function of doped carbon black. With doped carbon black dispersed in ethanol first, carbon black behaves together with ethanol like solvent. While it behaves closer to a dependent particle with doped carbon black directly added into aqueous solution, preventing the growth and aggregation of as-prepared particles. Therefore, it can be found that carbon black with different doping route has an important effect on the morphology of the fabricated MnO_2_.

**Fig 4:**
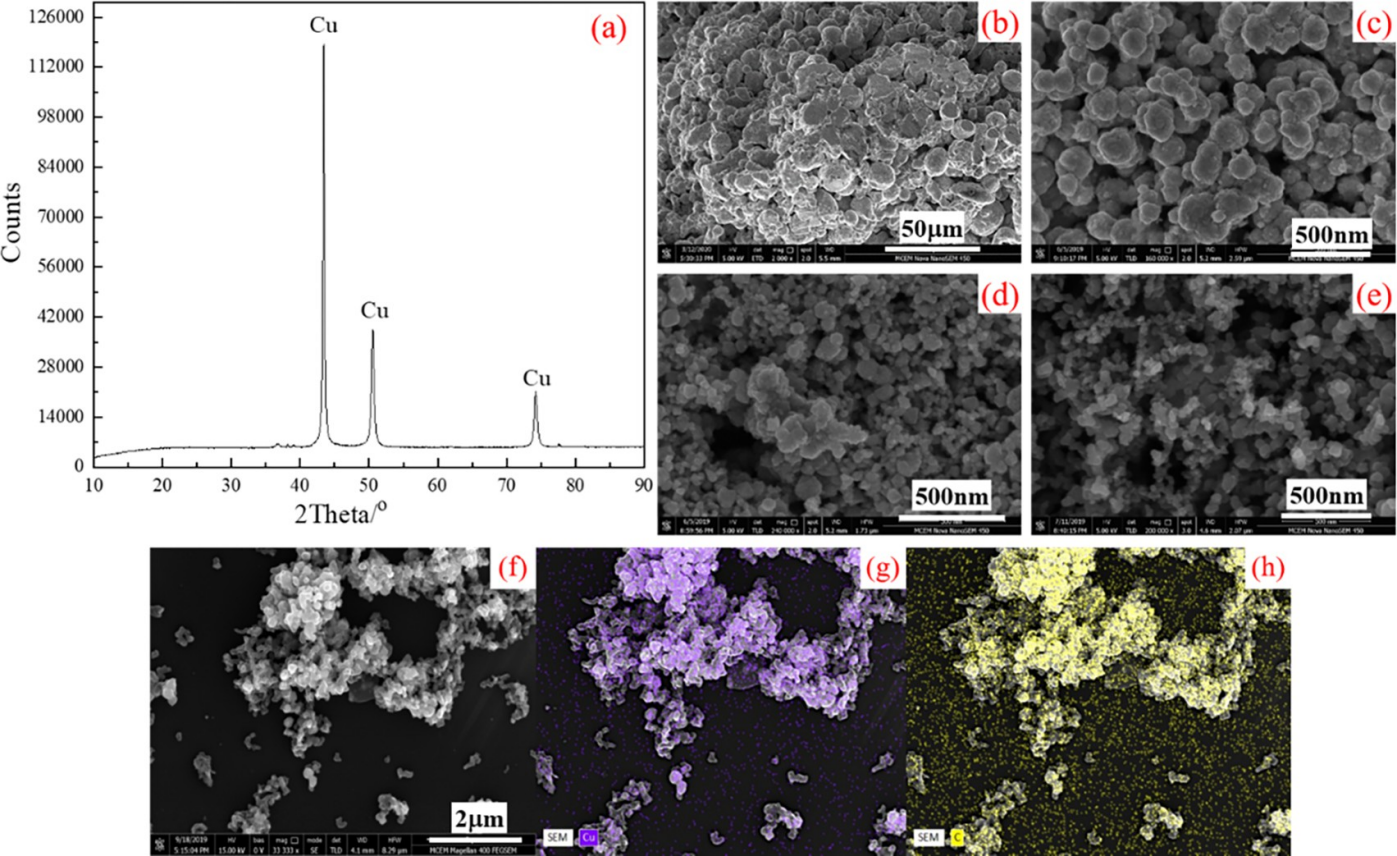
Characterization of various copper particles (a) typical XRD pattern of CCE; (b)-(e)SEM images of (b) commercial Cu (c) prepared Cu (d) CCD powder (e) CCE powder; and (f)-(h) SEM image and elements mapping of CCD powder.

Four types of copper powder including doping carbon black are further studied to investigate the effect of Cu on MnO_2_ morphology as shown in Fig 4. The typical XRD pattern of as prepared CCE is shown in Fig 4(A), it can be found that the characteristic peaks are closely matched with copper, indicating that the prepared Cu particles are pure without other impurities. SEM images of four types of copper particles are shown in Fig 4(B)–4(E), and it can be found that commercial copper powder is very dense, and the size of particles reaches tens of micro meters. In contrast, the average size of CCD and CCE is reduced to about 100nm as shown in Fig 4(D) and 4(E), and the particles are well dispersed compared with commercial copper. Especially for the product CCD, it can be found that there is distinguished space between particles with uniform size. These results reveal that the size of copper particles can be effectively reduced by doping carbon black, which may be owed to more nuclei sites during copper synthesis process.

To confirm that carbon black is successfully doped into copper particles, a typical SEM image of CCD particles and the corresponded elements mapping are shown in Fig 4(F)–4(H), wherein purple is assigned to Cu element, and yellow represents C element. From the elements mapping, it can be clearly seen that Cu and C elements are well-distributed in the Cu particles, and the distribution area overlaps completely, verifying the C is doped successfully on the surface of Cu particles.

From the above analysis, it can be found that MnO_2_ with good morphology can be obtained by using freshly prepared copper particles, which has smaller size and better dispersion. Besides, carbon black dispersed in ethanol in advance is facile to make the copper particle size more uniform, thus resulting in synthesis of nano-scaled MnO_2_.

**Fig 5:**
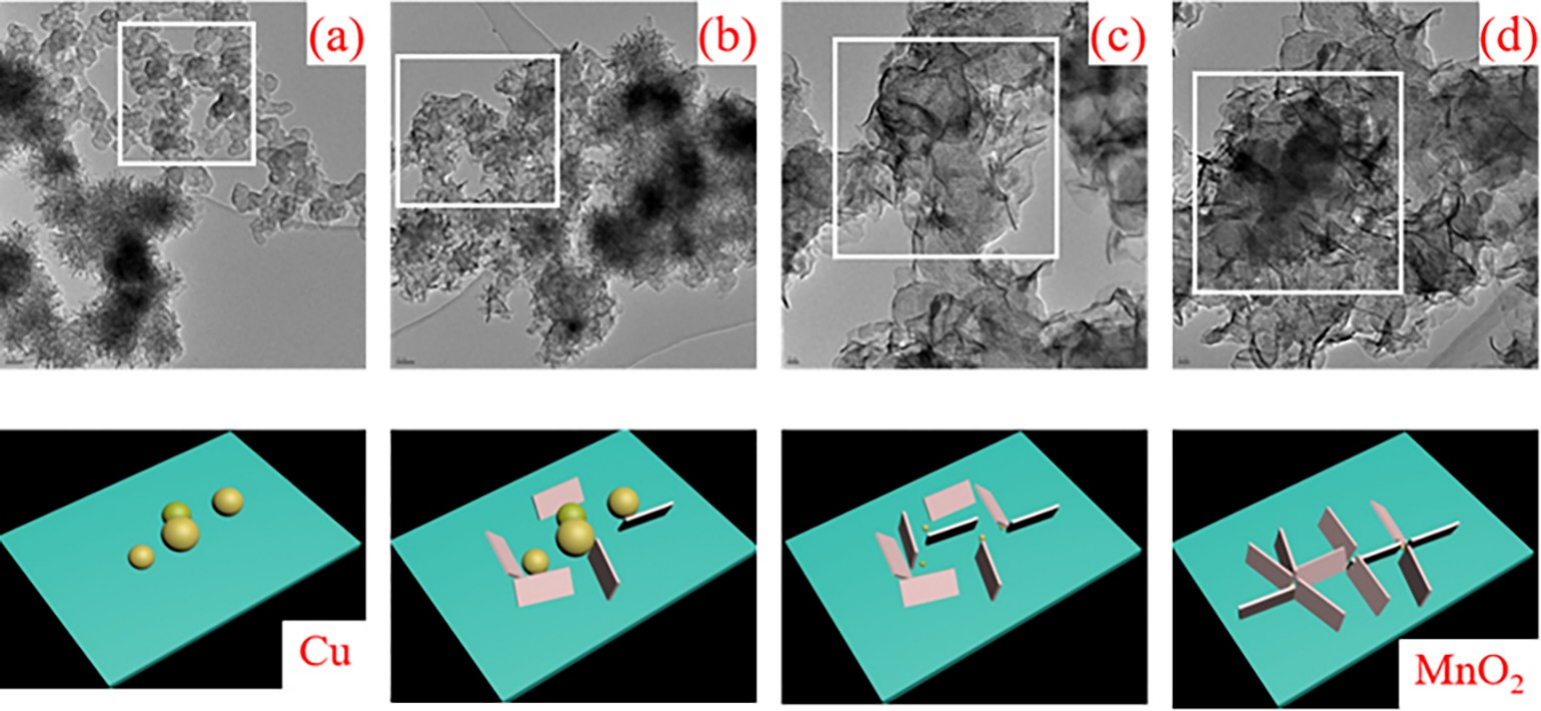
Growth process of nano flower MnO_2_: (a) original Cu particles; (b) a small part of copper particles take part in reactions and slices form; (c) most of the copper particles convert to slice MnO_2_; and (d) copper particles are consumed completely and slices assemble themselves, and flower-like shape is formed.

The growth mechanism of MnO_2_ is proposed via four steps as shown in Fig 5. First, copper particles act as the nuclei cores for the formation of MnO_2_ in Fig 5(A). When the reaction between Cu and KMnO_4_ occurs with the assist of H_2_SO_4_, and MnO_2_ nucleus was formed, then the process moves to step two in Fig 5(B). MnO_2_ nucleus grows to slice shape. Then, the process goes to step three with time prolongs as shown in Fig 5(C). In this step, most of the sphere particles transform to MnO_2_ slice, only a small percent of Cu particles is remained. Finally, copper particles are consumed, and MnO_2_ nucleus converts to slice completely. The slices are assembled together with Cu particles as the core, which leads to the formation of flower-like shape in rectangle area in Fig 5(B). They grow and aggregate gradually, and finally form nanostructure with a diameter of about 120 nm. Therefore, Cu particles are consumed, and flower-like nanostructure are formed, where the size and dispersity of copper particles play an important role in the morphology of fabricated MnO_2_.

### 3.2. Electrochemical property of the fabricated manganese dioxides

**Fig 6:**
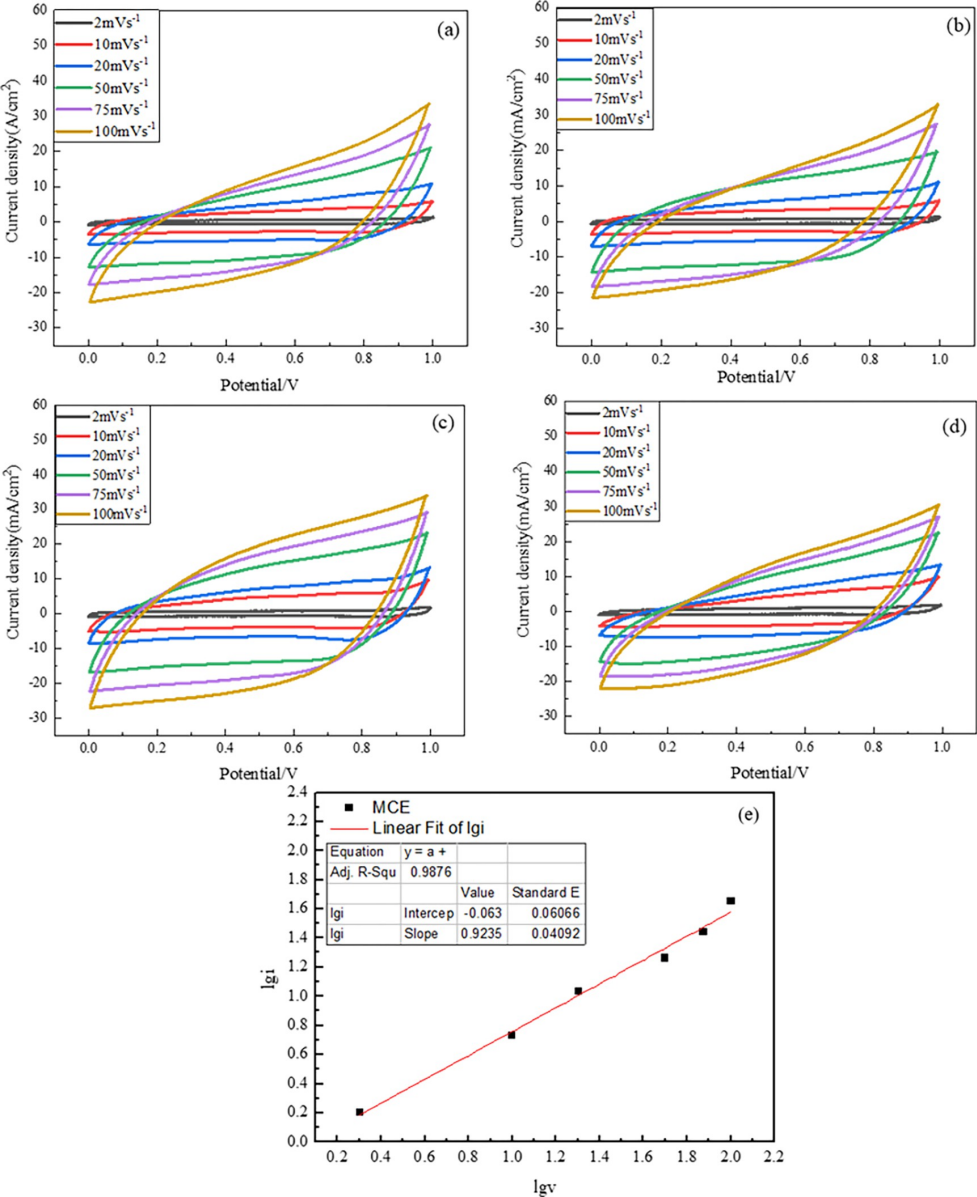
CV density curves collected for (a) product M (b) product MC (c) product MCD and (d) product MCE electrodes at various scan rates, (e) the typical calculated b value from CV curves for MCE.

To study the electrochemical performance of the manganese materials, electrochemical measurements are conducted in a three-electrode electrochemical cell, including CV curves and charge-discharge tests, where the fabricated MnO_2_ was used as the working electrode material. The collected cyclic voltammogram (CV) curves of the product M, MC, MCD and MCE at scan rates of 2 mV s^-1^, 10 mV s^-1^, 20 mV s^-1^, 50 mV s^-1^, 75 mV s^-1^ and 100 mV s^-1^ are compared in Fig 6. It can be found that CV curves show quasi-rectangular shapes, indicating the double layer capacitance of the electrode material. Especially, CV curves obtained at low san rate present double layer capacitance and good electrochemical reversibility. However, high scan rate causes the shape of CV curves a slight deviation from the rectangle shape, mainly due to the incomplete adsorption of electrolyte ions on the electrode surface caused by the polarization phenomenon. No redox peaks are observed in the curves at different scanning rates, suggesting that the electrode materials have pseudo capacitance property.

It can be found that the character of CV curves is similar with the geometric features of pseudo capacitance [39, 40]. Furtherly, b value can be used to evaluate the type of energy storage, herein, b value of 1 means a double layer type, and a range from 0.8 to 1 indicates the pseudo capacitance type [40, 41]. In our research, b values of 0.87, 0.91, 0.94 and 0.92 were achieved for M, MC, MCD and MCE electrode materials respectively. The calculated b value of MCE electrode material was shown in in Fig 6(E) as a sample. It can be found that all of the b values are bigger than 0.8, taking a further prove that the electrode materials prepared belong to pseudo capacitance type.

**Fig 7:**
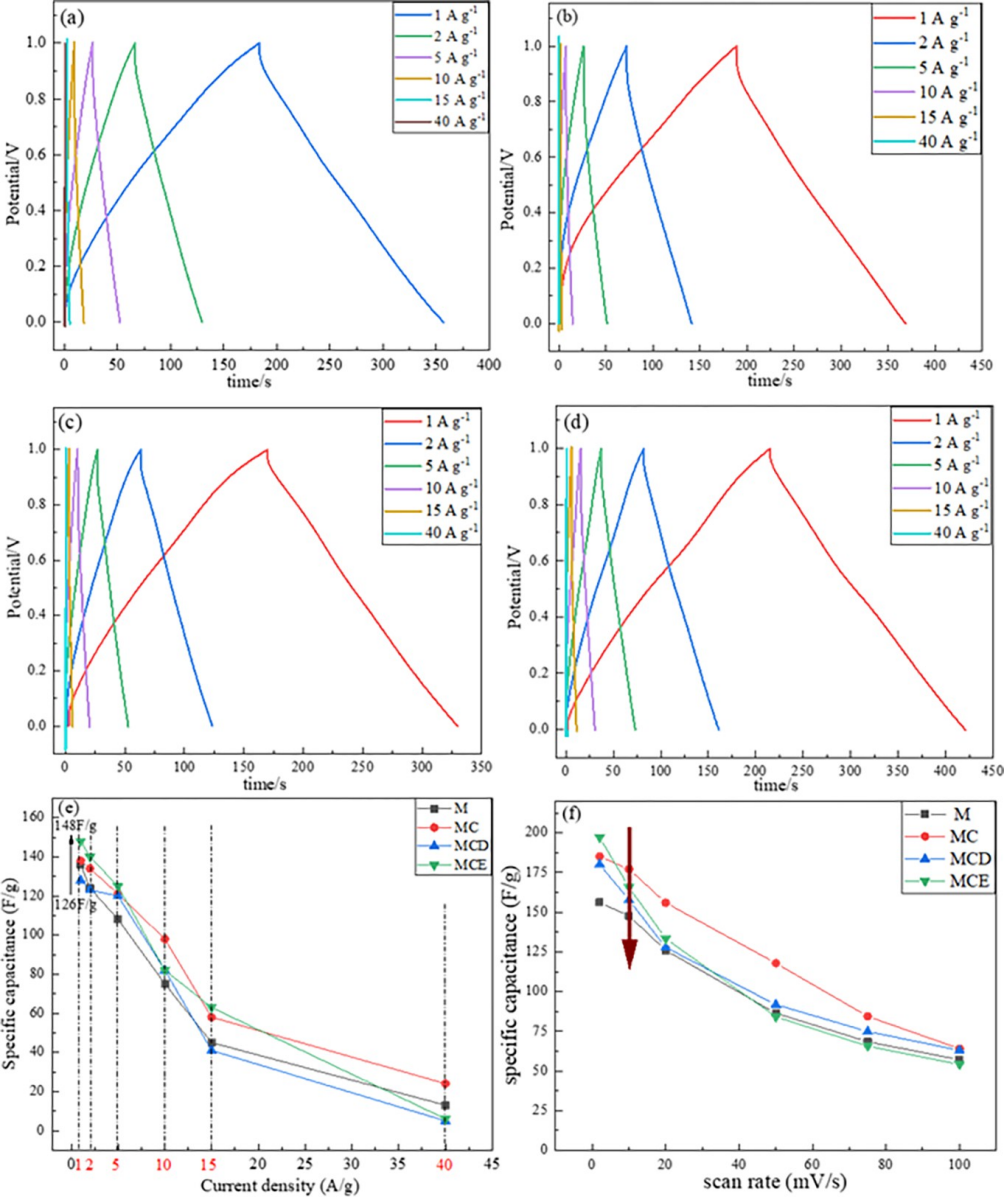
Charge/discharge curves of the electrodes at various current density: (a) product M (b) product MC (c) product MCD and (d) product MCE, (e) the specific capacitance at various current density, (f) specific capacitances of the fabricated M, MC, MCD and MCE at varied scan rates.

Charge—discharge curves of electrode material were obtained at different current densities (1 A g^-1^, 2 A g^-1^, 5 A g^-1^, 10 A g^-1^, 15 A g^-1^ and 40 A g^-1^) as shown in (Fig 7A–7D). The symmetry of the charge—discharge curves indicates that all the material electrodes have good electrochemical reversibility. Specific capacitance can be calculated according to the charge—discharge curves. From the calculated capacitance values from GCD as shown in Fig 7(E), it can be found that the MCE electrode material has the highest capacitance at small current density among four samples, achieving 148 F/g at 1 A/g. MC, MCD and MCE samples showed better electrochemical performance compared with M sample at low current density, proving the feasibility of the fabrication procedure with freshly prepared Cu particles. However, electrochemical performance dropped at high current density. Besides, specific capacitance also can be calculated from CV curves as shown in Fig 7(F), it can be found that the specific capacitance of the product MCE achieves the highest value of 197.2 F g^-1^ at 2mV s^-1^. Even for the worst performance, the value could reach 156.2 F g^-1^. With scan rates increasing, the product MC exhibits the superiority than other samples. The specific capacitance of the product MC achieved 177 F g^-1^ at 10mV s^-1^. It is known that the electrode materials show better specific capacitance performance at low scan rate, due to a higher transport efficiency of the electrons and ions [20, 27]. MnO_2_ prepared by using the CCE or carbon doped copper showed relatively high transport efficiency advantages among all of electrode materials due to a bigger gap between slices.

Table 1 shows the comparison of specific capacitance between this work and other similar reports. It can be found that the value of specific capacitance is quite closely with Zhao’s work [42, 43], and much higher than that of Li and Subramanian’s works [44, 45], where electrode active materials of single MnO_2_ was used. However, the electrochemical performance in not good enough when comparing with recent reports, using composites as electrode materials. Generally, a satisfied performance of MnO_2_-based electrode materials benefits from composite structure, the morphology and substrate electrode material. The comparison pushes us to improve and develop the as-prepared MnO_2_ in future, and composite structure based on the as-prepared MnO_2_ particles will be investigated.

**Table 1:** Comparison of specific capacitance with other studies on Ni substrate.

Composite	Substrate	Polymorph and morphology of MnO_2_	Specific capacitance	Ref.
MnO_2_	Ni foam	urchin-like nanoparticles	151.5 F/g at 1 A/g	[42]
	Ni foam	flower-like	197.3 F/g at 1 A/g	[43]
	Ni mesh	flower-like	168 F/g at 0.2 A/g	[44]
	Ni mesh	nanorods	166.2 F/g at 10mV/s	[45]
CNT@MnO_2_	carbon fibre paper	nanosheet	393.0 F/g at 0.25 A/g	[46]
Mn_3_O_4_/MnO_2_/N-doped graphene	Ni foam	nanosheet covered octahedrons	739.0 F/g at 0.5 A/g	[47]
MnO_2_	Ni foam	flower-like	148 F/g at 1 A/g; 197.2 F/g at 2 mV/s	this work

**Fig 8:**
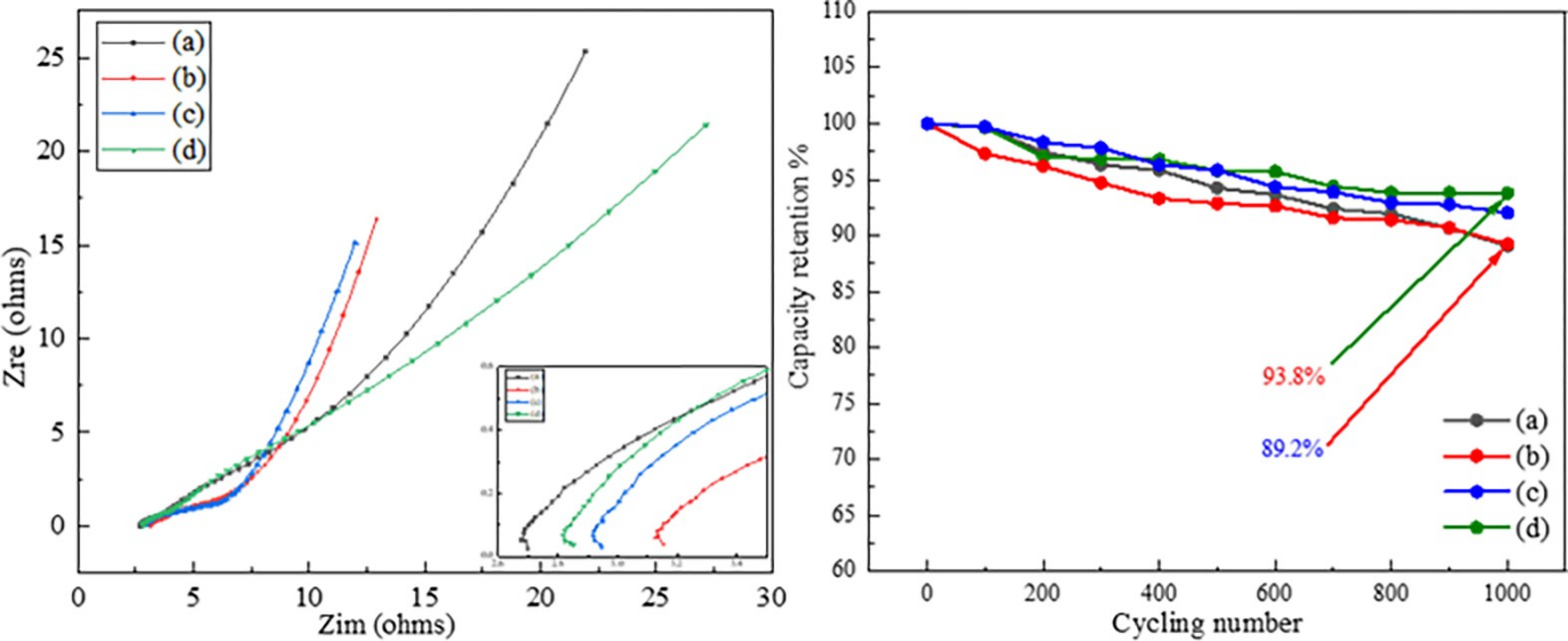
The Nyquist plots of the EIS of various electrode materials (left) and the cycling durability of various electrodes for 1000 cycles at 50 mV s-1 (right), wherein (a) product M (b) product MC (c) product MCD and (d) product MCE.

To understand of the electrochemical performance further, the test of electrochemical impedance spectroscopy (EIS) was carried out in the frequency range between 100 KHz–10 MHz as shown in Fig 8. The impedance plots are composed of a circle arc-like shape at high frequency and an oblique line at low frequency. It can be found that the line region at the low frequency shows an angle between 45^o^ and 90^o^ relative to the real axis, indicating the pseudocapacitance nature with diffusion control. At the high frequency, the intercept at real axis represents internal resistance (*R*_s_), including the combined resistance of the electrolyte, the electrode material and the contact resistance. The diameter of semicircle indicates the charge transfer resistance (*R*_ct_) at the interface, and the *R*_ct_ and *R*_s_ data collected from Fig 8 is shown in Table 2. It can be observed that MC, MCD and MCE electrode materials shows slightly higher *R*_s_ value than that of M electrode material (2.66 Ω), which should be caused by an increased contact resistance by doping carbon black. Moreover, it can be found that the *R*_s_ and *R*_ct_ value of MCE electrode material is small, indicating a superior property of conductivity and electron transfer. However, the angle of line region at the low frequency for MCE material is smallest, which shows an unsatisfied mass diffusion compared with other samples. Also, this result can be proved in Fig 7(E) and 7(F). It can be found that MCE electrode material shows excellent electrochemical property at low scan rate and small current density, which maybe owed to a smaller particle size. While, the specific capacitance of MCE electrode material drops heavily at high scan rate and current density, maybe attributing to a weak diffusion nature with scan rate and current density increasing, and this result matches well with the EIS data in line region.

**Table 2:** Data collected from EIS curves.

Sample	M	MC	MCD	MCE
*R*_ct_	0.06	0.08	0.05	0.04
*R*_s_	2.66	2.92	3.1	2.82

Cycle life is an important parameter for supercapacitor materials, and cycling durabilities of the electrode materials was measured as shown in Fig 8(B). It can be found that the capacitance retention of the product MCE electrode reaches 93.8% after 1000 cycles, which is the highest among all samples, However, the lowest capacitance retention with 89.2% is obtained from MC electrode material, resulting from an increased charge transfer resistance. Moreover, the result of cycling durability for four types of electrode materials matches well with the EIS data. The cycling results show that no significant structural changes are induced in the charge and discharge processes within the voltage window of 0 −1.0 V for 1000 cycles, suggesting that the prepared electrode materials are reliable candidates for pseudocapacitance reactions.

Based on the above results, it comes to a conclusion that the prepared M, MC, MCD, and MCE behave excellently as supercapacitor electrode, especially when prepared CCD and CCE copper particle is used. By using CCE and CCD with a smaller size, the electrochemical property of the fabricated electrode materials is improved significantly. It means that the particle size of the reactants has a crucial influence on the morphology of the resulting products. Moreover, it has influence on the performance of supercapacitor further, and this result has been described as shown in Fig 5. The size of the copper particles can be significantly reduced by directly doping carbon black, which could be caused by that doping carbon black increases the fresh nucleus. Moreover, when the carbon black is dispersed into ethanol, it behaves like solvent rather than solid particles together with ethanol. Therefore, when the reactions take place, carbon is closely contacted with produced copper particles. Otherwise, carbon is only doped in the synthesized copper particles, leading to bigger charge transfer resistance of MCD is compared with that of MCE. Therefore, MCE electrode material prepared by dispersing carbon black in ethanol shows better electrochemical performance. But both of MCD and MCE electrodes have larger contact resistances due to the doping carbon, which leads to the unsatisfactory specific capacitance at high scan rate.

Generally, there are two possible mechanisms for charge storage in MnO_2_ electrodes. One is the intercalation of Na^+^ cations during the redox process, and the other one is based on the adsorption of Na^+^ ions onto the surface of MnO_2_ particles [41]. In this study, the electrochemical processes are mainly based on the former one, which involve the insertion and deinsertion of Na^+^ cations from the electrolyte into the nanostructured MnO_2_. Due to the small size of prepared flower-like MnO_2_ nanostructure and large gaps between slices, the electron tunnels spaces are enlarged. Therefore, the electrochemical process on MnO_2_ nanostructure can be carried on more efficiently, leading to the better specific capacitance.

## 4. Conclusions

This research proved that four types of MnO_2_, including M, MC, MCD and MCE, were prepared by different copper sources in KMnO_4_-H_2_SO_4_ solution, and the synthesis of MnO_2_ via liquid phase method was suggested as a type of feasible route. The results showed that the size of copper particles had an important impact on the morphology of MnO_2_ powders as a result of copper acting as the nuclei centre during the formation-growth process. Electrochemical performance was measured using the obtained MnO_2_ as a working electrode material. The supercapacitor performance of the MCE materials electrode with a highest specific capacitance of 197.2 F g^-1^ at 2 mV s^-1^ and 148 F/g at 1 A/g was obtained. The results of CV curves and calculated b values showed that the electrode materials exhibited the character of pseudo capacitance. Moreover, EIS results showed that the values of charge transfer resistance in all materials electrode were smaller than 0.08 Ω. The cycling retention of MCE material electrode can reach 93.8% even after 1000 cycles. In general, this study may open a path for the synthesis of flowerlike MnO_2_ nanoparticles for achieving good performance in supercapacitors.

## Supporting information

S1 Data (ZIP)
